# Measurement Accuracy Enhancement via Radio Frequency Filtering in Distributed Brillouin Sensing

**DOI:** 10.3390/s19132878

**Published:** 2019-06-28

**Authors:** Cheng Feng, Stefan Preussler, Jaffar Emad Kadum, Thomas Schneider

**Affiliations:** THz Photonics Group, Institut für Hochfrequenztechnik, Technische Universität BraunschweigSchleinitzstr. 22, 38106 Braunschweig, Germany

**Keywords:** stimulated brillouin scattering, Brillouin optical time-domain analyzers, fiber optics sensors, distributed optical sensing, radio frequency filtering

## Abstract

In this article, we demonstrate the noise reduction and signal to noise ratio (SNR) enhancement in Brillouin optical time-domain analyzers (BOTDA). The results show that, although the main noise contribution comes from the Brillouin interaction itself, a simple low pass filtering on the detected radio frequency (RF) signal reduces remarkably the noise level of the BOTDA traces. The corresponding SNR enhancement depends on the employed cut-off frequency of the low pass filter. Due to the enhancement of the SNR, a mitigation of the standard deviation error of the Brillouin frequency shift (BFS) has been demonstrated. However, RF filters with low cut-off frequency could lead to distortions on the trace signals and therefore detection errors on a non-uniform BFS. The trade-off between the noise reduction and the signal distortion as well as an optimal cut-off frequency are discussed in detail.

## 1. Introduction

Distributed sensing applications such as the Brillouin optical time-domain analysis (BOTDA) have attracted immense interest in recent years in fields like health monitoring of large structures in oil and gas pipelines, railways and high voltage transmission lines [1,2], high temperature distributed measurement in industrial applications [3], distributed strain measurement for cracks detection and monitoring [4] and security intrusion monitoring over long fences [5]. BOTDA sensors exploit the stimulated Brillouin scattering (SBS) in optical fibers, that initiates after a pulsed pump wave counter-propagates with a probe continuous wave (CW) which leads to an excitation of an acoustic wave. The SBS interaction leads to a frequency down-shifted gain region where a counter propagating wave is amplified and a frequency up-shifted loss region where the counter propagating wave is attenuated. Based on this interaction, the energy is transferred from the higher frequency pump wave to the probe wave via the acoustic wave, leading to a depletion of the pump signal and a Brillouin gain for the probe signal. The efficiency of this energy transfer depends on the frequency offset between the pump and probe wave, which is called Brillouin frequency shift (BFS) and around 11 GHz in standard single mode fibers (SSMF) [6]. This BFS is sensitive to both temperature and strain in the optical fiber, which makes the basic principle of the BOTDA [7]. In order to accurately estimate the BFS, the Brillouin gain spectrum (BGS) is measured at each fiber location by sweeping the pump or probe frequency offset [7] and reconstructed by fitting with the theoretical profile [8,9]. However, the gain in a pulse-CW interaction is limited due to the short interaction length, which is determined by the spatial resolution requirement and reduces the signal to noise ratio (SNR) of the detected probe signal drastically. Since for a conventional BOTDA system, i.e., single pulse pump wave, single sideband probe CW without fiber loop configuration as in the first experimental demonstration [10] and defined as the standard scheme in [11], the pump wave is constrained by modulation instability (MI) [12] and the probe power is limited by nonlocal effects [13], the power of the pump and probe wave cannot be increased to enhance the SNR.

The accuracy of the BFS estimation is the key performance of the BOTDA sensing. The estimation error in the experiment can be statistically determined by the standard deviation of the measured BFS after a large number of consecutive measurements. It depends on the full-width at half maximum (FWHM) of the BGS $\Delta \nu_{B}$, the frequency scanning step δ and the system noise σ at the location of the fiber section, which is the inverse of the system SNR [11]. Therefore, noise reduction has a direct impact on the enhancement of the accuracy of the BFS estimation.

Many methods have been proposed to increase the SNR in BOTDA sensors. The SNR can be enhanced by increasing the number of averages of the data traces, however, this comes at the cost of an increased measurement time. Other methods like differential π-phase-shifted pulse pair [14], Raman amplification [15], self-heterodyne detection [16], optical pulse coding [17], wavelet de-noising techniques [18] and 2D and 3D image restoration [19] have been proposed as well. However, these methods complicate either the experimental setup or the post-processing algorithm. Here, a very simple and direct method for the noise reduction in the detected probe signal, based on radio frequency(RF) low pass filtering, is presented. Since the thermal, shot and dark noise from a detector, and the relative intensity noise (RIN) from the laser source [16,20], are proportional to the square root of the system bandwidth $B_{sys}$ [21], RF low pass filtering could theoretically suppress these noise contributions in a BOTDA system by a factor of $\sqrt{B_{sys}/B_{f}}$, where $B_{f}$ is the filter bandwidth. However, the main noise contribution in a Brillouin amplifier comes from the Brillouin interaction itself [22,23]. This contribution cannot simply be filtered out. However, as we will show in the following, a simple filter bandwidth reduction has a significant effect on the BOTDA sensor performance.

## 2. Simulation

### 2.1. Theory and Modeling

For a CW-SBS interaction, the complex BGS profile is given by [24]

(1) $$G_{B}\left(\omega \right)=\frac{\frac{1}{2}g_{0}P_{p}}{1-2j\left(\omega -\omega_{B}\right)/\Gamma_{B}}$$

where $g_{0}$ is the SBS gain coefficient, $P_{p}$ is the pump power launched into the fiber, $\omega_{B}/2\pi$ is the BFS and $\Delta \nu_{B}=\Gamma_{B}/2\pi$ is the SBS gain bandwidth, which is usually in the range of 20 to 30 MHz in SSMF at 1550 nm pump wavelength and can be reduced by one order of magnitude with several methods for CW-SBS interactions [25,26,27,28]. The imaginary part of Equation (1) is the Brillouin phase spectrum and the real part represents the BGS which is approximated by a Lorentzian shape [24]. Provided that the pump depletion is negligible and the extinction ratio (ER) of the pump pulse is infinitely high so that no pump leakage-probe interaction is considered [29,30,31], the local Brillouin gain $g_{B}$ that will be experienced by the probe wave at position *z* is given by

(2) $$g_{B}\left(\omega ,z\right)=\exp\left[G_{B}\left(\omega \right)L_{eff}\right]\cdot \exp\left[-2\alpha z\right]$$

where α is the linear loss coefficient of the fiber, $L_{eff}=\left[1-\exp\left(-\alpha L\right)\right]/\alpha$ represents the effective length, $L=v_{c}T/2$ has the same expression as the spatial resolution, where $v_{c}$ is the speed of light in the fiber and *T* is the pulse width.

In BOTDA sensors, a pulse train is launched into the fiber as the pump signal with a pulse width *T* and peak pulse power $P_{p}$. Thus, each pump pulse interrogates the fiber sections sequentially and consequently, the CW probe wave at different fiber sections will experience different Brillouin gain, generating time resolved traces, according to the pulse train repetition rate. Then the frequency domain representation of the detected BOTDA signal is obtained by applying a Fourier transformation (FT) to the detected BOTDA traces. Please note that, the FT of the BOTDA probe signal in the frequency domain determines the required bandwidth of the photodiode (PD) while the BGS is scanned by the pump-probe frequency difference.

In an additive white Gaussian noise channel (AWGN), the noise power is directly proportional to the bandwidth. Thus, noise reduction and SNR enhancement can be performed by removing (filtering) noisy spectral components out of the detected RF spectrum of the probe signal. Let H(ω) be the filter transmission response, $P_{s}\left(\omega \right)$ the FT of the time-varying probe traces and $P_{out}\left(\omega \right)$ the RF filtered output spectrum, then

(3) $$P_{out}\left(\omega \right)=P_{s}\left(\omega \right)H\left(\omega \right)$$

The filtered time domain probe traces can be obtained by applying the inverse FT to $P_{out}\left(\omega \right)$

(4) $$P_{out}\left(t\right)=F^{-1}\left[P_{out}\left(\omega \right)\right]$$

where $F^{-1}$ denotes the inverse FT.

### 2.2. Frequency and Time Domain Simulation

**Figure 1:**
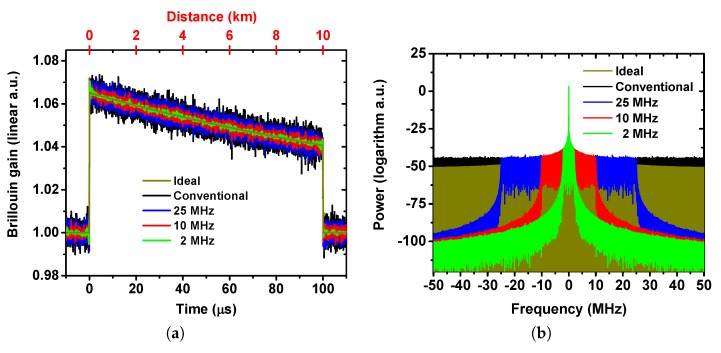
(**a**) Simulated time domain traces of the ideal, conventional and filtered probe signal; (**b**) simulated spectrum of the detected radio frequency (RF) ideal, conventional and filtered probe signal with different filter bandwidths.

The simulation is carried out based on the simple model presented in Section 2.1. In BOTDA, the Brillouin gain experienced by the probe signal is calculated according to Equations (1) and (2) at each fiber section. The BGS is calculated with 200 MHz frequency span and 1 MHz step. The repetition rate and pulse width of the pump pulse in the simulation are 8.3 kHz and 100 ns, respectively (same values as for the experiment). The spatial resolution Δz is 10 m which is determined by the pump pulse width(100 ns) and is given by $v_{c}T/2$, where $v_{c}$ is the speed of light in the fiber and *T* is the pulse width. The probe and pump power are set to −14 dBm and 18 dBm, respectively. In order to study the effect of the RF filtering, random noise is added to the traces. For a fair comparison, the noise level on all the traces in the simulation are the same. The spectra of the traces are obtained by applying a fast FT to the time domain traces. The filtering effect is simulated by multiplying the spectra with the transfer function of a RF low pass filter. The filtered time domain traces are retrieved by applying inverse fast FT to the filtered spectrum. The SBS linewidth is 50 MHz, while the assumed BFS is 10.615 GHz and the total fiber length is 10 km. Figure 1a shows the time domain traces of the probe wave for the ideal case, i.e., without any noise, for the conventional case, i.e., with noise (it refers to a direct detection at the full detector bandwidth without employing any additional filters in the experiment), and for low pass filtered cases with cut-off frequencies of 2, 10 and 25 MHz. As it is clearly illustrated, the low pass filters reduce the noise, i.e., the lower the filter bandwidth is, the lower the noise will be.

The ideal spectrum in Figure 1b represents the frequency spectrum of the detected RF trace signal without any noise (dark yellow curves in both Figure 1a,b) while the black one represents the spectrum of a conventional BOTDA signal after applying noise but without employing any filtering. As expected, using low pass filtering rejects the out-of-band noise components and therefore reduces the noise of the time domain traces.

### 2.3. Measurement Accuracy

**Figure 2:**
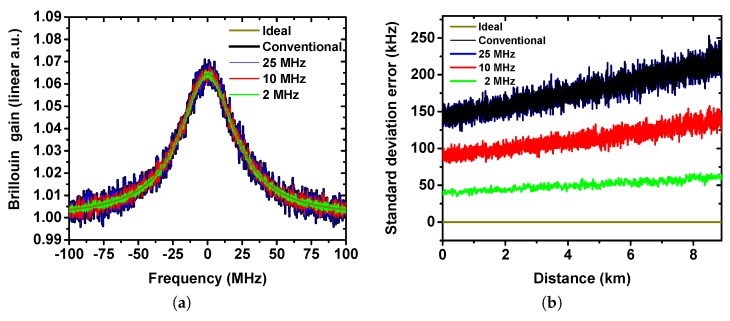
(**a**) Reconstructed Brillouin gain spectrum (BGS) (resolution 0.1 MHz) at the near end of the fiber and (**b**) standard deviation error after applying RF filtering with different bandwidths in the simulation.

The measurement accuracy, which plays a vital role in the evaluation of sensor performance, symbolizes the uncertainty of the measurand (temperature or strain). It is characterized by the BFS estimation error in a BOTDA system and highly dependent on the probe SNR. As the reconstructed BGSs in Figure 2a depicts, the BGS with a lower bandwidth filter is clearer and makes the BFS estimation easier, which shows coherence to the time domain trace results in Figure 1a. If the noise is defined as the the root mean square (RMS) amplitude of the BGS in the frequency domain [11], the noise level for the 2 MHz, 10 MHz and 25 MHz filter are 29.8%, 64.1% and 99.8% compared to the conventional case. Since the pulse width in the simulation is 100 ns and hence its BGS is more Lorentzian-like [32], the BFS along the fiber is reconstructed by a Lorentzian fitting of the measured BGS. In the simulation, the standard deviation is calculated from a set of 200 BFS measurements at each fiber segment. Figure 2b illustrates the standard deviation error. The ideal trace, which is considered without any noise, has a zero standard deviation error, while the curve with too large bandwidth (with noise but without applying proper low pass filtering) has the highest standard deviation error. Note that the conventional error curve and the curve after applying the 25 MHz filter are well overlapped, since the 25 MHz filter allows still higher noise frequency components and thus lower SNR. The standard deviation error is decreased when the cut-off frequency of the filter is reduced. The standard deviation error at the end of the fiber with 2 MHz filtering is about only 50 kHz compared to 210 kHz when a 25 MHz cut-off frequency is used. Since the pump power is reduced exponentially along the fiber due to the fiber losses, the standard deviation error is increased exponentially.

## 3. Experiment and Results

### 3.1. Experimental Setup

**Figure 3:**
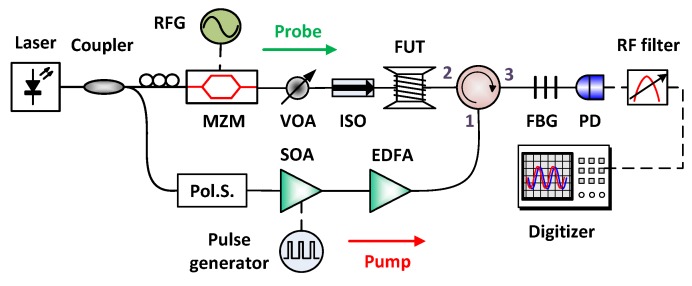
Experimental setup: RFG: radio frequency generator, MZM: Mach Zehnder modulator, VOA: variable optical attenuator, ISO: isolator, FUT: fiber under test, CIR: circulator, FBG: fiber Bragg grating, Pol.S: polarization scrambler, SOA: semiconductor optical amplifier, EDFA: Erbium-doped fiber amplifier, PD: photodiode.

Figure 3 shows the experimental setup, the output from the DFB-laser at 1550 nm is split into two branches via a 90/10 optical coupler. In the lower branch, the optical pulses are generated using a switching type semiconductor optical amplifier (SOA) (Thorlab SOA1013SXS switched by a Highland Technology T160-9 driving board) driven by an electrical pulse sequence with 8.33 kHz repetition rate to get high ER (32 dB) optical pulses. The optical pulses are amplified by an erbium-doped fiber amplifier (EDFA) (Photop Koncent, Inc. PTEDFA-PA-C-SCH-15-FC/APC with constant current mode) to 14 dBm to avoid MI [12]. Though the pump power is not optimized to the best performance, a better frequency error improvement could be demonstrated with this power level. To compensate the polarization fading, a polarization scrambler (General Photonics PSY-101) was used before directing the pump pulses into the fiber via an optical circulator. In the upper branch, the double-side band probe wave was formed by modulating the laser via a Mach Zehnder modulator(MZM) driven by an RF generator (Anritsu MG3692C), which scans the RF frequency with a span of 150 MHz and 1 MHz step to reconstruct the BGS. The bias point of the MZM was set to get a probe signal with double sideband and suppressed-carrier to generate dual (gain and loss) Brillouin interaction that compensates each other to reduce pump depletion [33]. The power of the probe wave was set to −14 dBm to avoid nonlocal effects [13]. After SBS interaction between pump and probe waves, the BOTDA signal was filtered by a narrowband Fiber Bragg Grating (FBG, AOS T-FBG) to remove the upper sideband of the probe wave. The RF probe signal was detected by a PD (Optilab PD-20)—with an electrical bandwidth of 20 GHz and a responsitivity of 0.85 A/W—and passed through a commercial low pass filter. After that, the probe signal was received by a digitizer (Acqiris U5309A, 300 MHz bandwidth) with a sample rate of 1 GSa/s and 8 bits resolution and computer for averaging and further analysis.

### 3.2. Results

**Figure 4:**
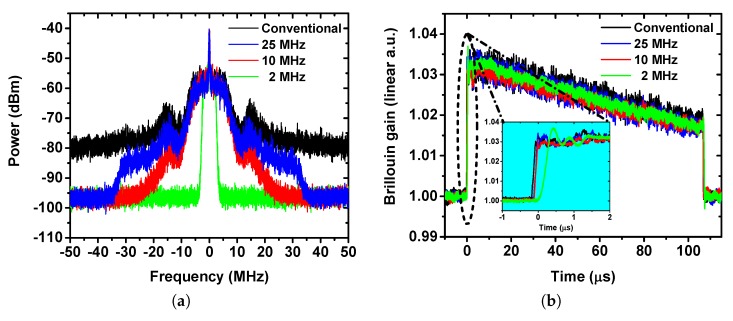
Experimental measurement results using RF low pass filtering: (**a**) RF spectrum measured by ESA; (**b**) time domain traces measured by a digitizer. The inset is the zoom in of the trace at the near end of the fiber.

First, we study the effect of the RF filtering on the time domain traces in regard to noise reduction, Figure 4a illustrates the RF spectra measured by an electrical spectrum analyzer (ESA). As it can be seen, the spectrum is reduced depending on the cut-off frequency of the used low pass filter. Figure 4b shows the corresponding time domain traces measured by a digitizer, the effect of RF filtering can be seen from the noise level mitigation on these traces. According to the conventional definition of RMS noise in the frequency domain [11], the noise level for 2 MHz, 10 MHz and 25 MHz are 65.7%, 78.9% and 94.7% compared to the conventional case, respectively. As clearly demonstrated, the noise suppression via low pass filtering in the experiment is, though remarkable, lower than in the simulation, especially for low filter bandwidths (2 MHz). This indicates that, the noise in the BOTDA system is not a pure AWGN channel. With a simple filter, the bandwidth dependent AWGN can be efficiently suppressed but the gain dependent SBS noise cannot.

**Figure 5:**
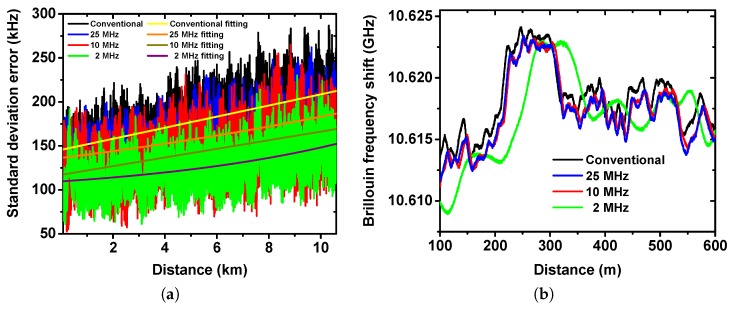
Experimental measurement using RF lowpass filtering: (**a**) Standard deviation error versus distance, solid lines represent the exponential fitting; (**b**) Detection of the strain point.

Next, the effect of the RF filtering on the BOTDA accuracy was evaluated. Figure 5a shows the evolution of the standard deviation error along the fiber. Due to the noise reduction and SNR enhancement, the impact of the RF filter can be clearly observed. The error is 208 kHz at 10 km distance with a 25 MHz filter, which corresponds to a measurand (temperature) resolution of 0.2 ${}^{{}^{\circ}}$C, while it is reduced to 147 kHz (measurand resolution 0.15 ${}^{{}^{\circ}}$C) with a 2 MHz filter. Meanwhile, applying 2 MHz filtering reduces the standard deviation error by 32% compared to the conventional case.

At last, the figure of merit (FoM) [11] as a criteria of the sensing performance is calculated to be 0.0277, 0.0316, 0.0348 and 0.0387 for the conventional case and for the applied filter bandwidth of 25 MHz, 10 MHz and 2 MHz, respectively. As clearly demonstrated, a narrower bandwidth filter leads to a lower frequency error and thus a better sensing performance (higher FoM). Since an optimized pump power (at the MI threshold) would achieve a higher SNR and hence a further lower frequency error, a higher FoM value would be expected with an optimized pump power.

## 4. Discussion

To further asses the performance of BOTDA with RF filtering, a strain point measurement was conducted. As illustrated in Figure 5b, for all types of filters, the sensor detects the strain point in the near end. However, if the filter bandwidth is too low, as shown by the 2 MHz trace, the detected location of the strain point is shifted, while for the cases with 10 and 25 MHz filter, the trace is almost equally undistorted as that for the conventional case. This shift comes from the group delay at the frequency edge of the applied low pass filter. For a high bandwidth filter, only a few frequency components of the trace signal are at the edge of the filter, thus the group delay is negligible and vice versa. According to the data sheet, the 2 MHz filter provides a group delay of about 500 ns at its edge, which agrees very well with the strain spot shift.

Under the criteria that the step response of the filter should not distort the temporal response of the sensing system, the optimal RF filter bandwidth can also be theoretically estimated. For a BOTDA system with 10 m spatial resolution achieved by 100 ns pump pulse, the best noise performance with the lowest trace distortion is optimized with a filter rising time of 1/3×100≈33 ns [34]. Since the relationship between the bandwidth *B* and the rising time $t_{r}$ for a typical low pass filter is approximately $t_{r}\cdot B\approx 0.35$ [35], the optimal filter bandwidth can be calculated as 10 MHz, which shows very good agreement to our simulation as well as experimental results. A filter with a lower bandwidth will further increase the noise performance (clearer traces), but at the cost of trace distortion and measurement error. Therefore, the cut-off frequency has to be selected carefully to balance between noise performance and trace distortion.

Since the bandwidth of the digitizer is narrower than the detector in the experiment, the system bandwidth $B_{sys}$ is limited to 300 MHz by the analog-to-digital conversion (ADC) process. For a pure AWGN channel, the remained noise level normalized to the conventional case could theoretically reach 8.16%, 18.26% and 28.87% for a 2 MHz, 10 MHz and 25 MHz filter, respectively. However, there are numerous practical factors that prevent the noise suppression being that high. The most important factor is that, in a Brillouin amplifier the main noise contribution comes from the Brillouin interaction itself [22,23]. Instead of evenly distributed in system bandwidth, the SBS noise power is gain dependent [22] and thus cannot be significantly reduced with a simple filter. It is believed that the influence of the SBS noise in a distributed Brillouin sensing system, especially with a long pulse width as in the experiment, can hardly be neglected and is of great significance for further investigations. Other factors that limit the noise suppression are not enough samples in the BGS to make the RMS calculation statistically accurate, the steepness of the filter profile, etc.

## 5. Conclusions

In this article, we have presented RF filtering on the detected signal to mitigate the noise level and SNR enhancement in BOTDA sensors. We have theoretically and experimentally demonstrated that the noise level can be reduced by low pass filters, and consequently, the measured standard deviation error of the BFS is reduced. The noise reduction level depends on the employed cut-off frequency of the low pass filter. However, especially for low bandwidths, the noise suppression is not as high as theoretically possible for a pure AWGN channel. This clearly shows the influence of the gain dependent SBS noise on BOTDA sensors, which requires further in detail investigation. The results also indicate that RF filters with too low cut-off frequency lead to a distortion of the trace signals and therefore detection errors for a non-uniform BFS. Instead of an RF filter, a cost effective detector with optimal bandwidth can be used.
